# Clinical and Biological Activity of Chemoimmunotherapy in Advanced Endometrial Adenocarcinoma: A Phase II Trial of the Big Ten Cancer Research Consortium

**DOI:** 10.1158/2767-9764.CRC-22-0147

**Published:** 2022-10-28

**Authors:** Emma L. Barber, Siqi Chen, Mario Javier Pineda, Sharon E. Robertson, Emily K. Hill, Deanna Teoh, Jeanne Schilder, Kaitlyn L. O'Shea, Masha Kocherginsky, Bin Zhang, Daniela Matei

**Affiliations:** 1 Department of Obstetrics and Gynecology, Northwestern University Feinberg School of Medicine, Chicago, Illinois.; 2 Robert H Lurie Comprehensive Cancer center, Chicago, Illinois.; 3 Department of Medicine, Northwestern University, Chicago, Illinois.; 4 Ironwood Cancer and Research Centers, Gilbert, Arizona.; 5 Department of Obstetrics and Gynecology, Indiana University, Indianapolis, Indiana.; 6 Department of Obstetrics and Gynecology, University of Iowa Hospitals and Clinics, Iowa City, Iowa.; 7 Department of Obstetrics and Gynecology, University of Minnesota, Minneapolis, Minnesota.; 8 Department of Preventive Medicine, Northwestern University, Chicago, Illinois.

## Abstract

**Purpose:**

The objective of this study was to assess the efficacy and safety of pembrolizumab in combination with standard carboplatin/paclitaxel in patients with advanced endometrial cancer.

**Patients and Methods:**

This single-arm, open-label, multicenter phase II study enrolled patients with RECIST measurable advanced endometrial cancer. Patients could have received ≤ 1 prior platinum-based regimen and ≤ one non-platinum chemotherapy. The primary endpoint was objective response rate (ORR). Planned sample size of 46 subjects provided 80% power to detect 15% ORR improvement compared with historical control rate of 50%.

**Results:**

A total of 46 patients were enrolled and 43 were evaluable for ORR. Median age was 66 (range: 43–86). Thirty-four (73.9%) patients had recurrent and 12 (26.1%) primary metastatic endometrial cancer. Patients received carboplatin AUC6, paclitaxel 175 mg/m^2^, and pembrolizumab 200 mg i.v. every 3 weeks for up to six cycles. ORR was 74.4% (32/43), higher than historic controls (*P* = 0.001). Median progression-free survival (PFS) was 10.6 months (95% confidence interval, 8.3–13.9 months). The most common grade 1–2 treatment-related adverse event (TRAE) included anemia (56.5%), alopecia (47.8%), fatigue (47.8%), and neuropathy (13%), while the most common grade 3–4 TRAEs were lymphopenia, leukopenia, and anemia (19.6% each). High-dimensional spectral flow cytometry (CyTEK) identified enrichment in peripheral CD8^+^ and CD4^+^ T-cell populations at baseline in responders. The CD8^+^ T-cell compartment in responders exhibited greater expression levels of PD-1 and PD-L1 and higher abundance of effector memory CD8^+^ cells compared with nonresponders.

**Conclusions:**

Addition of pembrolizumab to carboplatin and paclitaxel for advanced endometrial cancer was tolerated and improved ORR compared with historical outcomes.

**Significance:**

The results of the study support that the combination of pembrolizumab with carboplatin and paclitaxel is well tolerated and active in patients with advanced endometrial cancer. The duration of response and the PFS were significantly longer in patients with mismatch repair deficient/microsatellite instability-high compared with mismatch repair proficient/microsatellite stable tumors. Responders to treatment tend to have enriched CD8^+^ T-cell and CD4^+^ T-cell populations among peripheral blood mononuclear cells at baseline.

## Introduction

Endometrial cancer is the most common cancer of the female gynecologic tract and the fourth most common cancer in the United States (1). Both incidence and disease-related mortality are rising (2–4). It is estimated that over 65,000 women will be diagnosed with endometrial cancer and nearly 13,000 will die of the disease annually (5). Although early-stage endometrial cancer has a favorable prognosis, approximately 20%–25% of patients are diagnosed with advanced stage disease. Their prognosis is poor, with a 5-year survival of 18%. Standard-of-care therapy for this patient population consists of carboplatin and paclitaxel (6). The objective response rate (ORR) to first-line chemotherapy in metastatic endometrial cancer ranges from 38% to 52% and the median progression-free survival (PFS) ranges from 8 to 13 months (6, 7).

New strategies targeting immune checkpoints have impacted the outcomes of several tumor types (melanoma, lymphoma, renal, lung, bladder cancer) garnering FDA approval for a wide spectrum of malignancies (8–10). PD-1 signaling blocks T-cell activation keeping nascent T cells in check and preventing immune responses against normal tissues. During cancer progression, this immune inhibitory pathway is activated when PD-1 ligands (PD-L1 and PD-L2) are upregulated on either tumor or immune cells in the tumor microenvironment (TME) and permits evasion from antitumor immune surveillance (11). PD-1–targeted therapy removes this block, allowing the immune system to attack and eliminate tumor cells (12). Pembrolizumab, a mAb that targets the programmed death receptor-1 (PD-1), is approved for treatment of solid tumors that are microsatellite unstable (MSI-H) or mismatch repair deficient (dMMR), independent of tumor site of origin (13, 14). Up to 16% of recurrent endometrial tumors are MSI-H/dMMR (15) and thus eligible for treatment with pembrolizumab. Response rates in this group are as high as 57% (16). In contrast, the efficacy of pembrolizumab in patients with microsatellite stable (MSS) or mismatch repair proficient (pMMR) endometrial cancer is low, with ORR of 13% (17). This led to high interest in developing new combination regimens that would increase the efficacy of immune check inhibitors in this setting. The combination of pembrolizumab and lenvatinib was recently approved for the treatment of recurrent MSS uterine cancer, based on favorable progression-free and overall survival (PFS and OS) in KEYNOTE-775 trial compared with physician's choice chemotherapy (18, 19). These recent observations underscore the higher propensity of response to immunotherapy in uterine cancer, compared with other gynecologic tumors.

Chemotherapy has been shown to be synergistic with immunotherapy in other tumor types (20, 21). Chemotherapy can induce immunogenic cell death and disrupt the strategies that tumors use to evade the body's immune response (22). Cell death induced by chemotherapy results in the release of tumor antigens and of danger-associated molecular patterns (DAMP) into the TME, which in turn can trigger an inflammatory reaction (23, 24). When combined with PD-1 or PD-L1 inhibitors, chemotherapy has been shown to augment the presentation of tumor-specific antigens and to enhance the cytotoxic T-cell antitumor responses. Thus, immunochemotherapy combinations have resulted in increased clinical efficacy in other malignancies, such as lung cancer (20, 25). However, in ovarian cancer, addition of immunotherapy to standard chemotherapy has not resulted in improved clinical outcomes (26, 27).

Given these considerations, there is high interest in evaluating the clinical activity of chemoimmunotherapy in metastatic endometrial cancer. Here we report results of a multi-institutional phase II trial testing the effects of pembrolizumab in combination with carboplatin and paclitaxel for patients with advanced or recurrent endometrial cancer. The primary objective of the study was to estimate the ORR induced by pembrolizumab in combination with standard carboplatin/paclitaxel in this patient population. Secondary objectives included determining the toxicities of the regimen and evaluating potential biomarkers associated with response in tumor and in peripheral circulation.

## Materials and Methods

### Study Design and Patients

This was a single-arm, single-stage, open-label, multicenter, phase II trial coordinated by the Big Ten Cancer Research Consortium (BTCRC). Eligible patients had metastatic endometrial cancer, either newly diagnosed or recurrent. Patients must have had definitive surgery including at least a hysterectomy with bilateral salpingo-oophorectomy. All epithelial histologies were eligible for participation; carcinosarcoma was excluded. Patients were required to have measurable disease, according to RECIST v1.1. Disease in a radiated field as the only site of disease was considered acceptable provided there was clear progression after completion of radiotherapy. Treatment consisted of carboplatin (AUC6 IV), paclitaxel (175 mg/m^2^ i.v.), and pembrolizumab (200 mg i.v.) given every 3 weeks for up to six cycles, no maintenance pembrolizumab was used. Decreased dosing of carboplatin (AUC5) and paclitaxel (135 mg/m^2^) was permitted for patients with a history of prior pelvic radiation or prior platinum-based therapy. The first 6 patients were enrolled in a safety lead-in cohort to determine tolerability of the regimen, because this combination had not been tested in endometrial cancer. If >1 dose-limiting toxicities (DLT) were to be recorded among the first 6 treated patients, then the safety cohort would be expanded to 6 additional subjects. DLTs were defined as any grade 4 non-hematologic toxicity, any grade 3 non-hematologic malignancy lasting >3 days despite best supportive care, sepsis requiring intravenous antibiotics, grade 3 or 4 febrile neutropenia, thrombocytopenia <25,000/mm^3^ associated with bleeding event or necessitating >2 weeks delay in further treatment, any grade 5 toxicity, or dose modifications occurring during the first two cycles of treatment.

Patients may have received up to one prior platinum containing regimen and up to one non-platinum chemotherapy regimen. For those patients who had received prior platinum therapy, a disease-free interval of greater than 6 months after completion of carboplatin was required. Prior radiotherapy as well as hormonal or biologic agents were permitted if they were discontinued at least 28 days prior to starting protocol therapy. All toxicities from prior therapy had to be resolved to ≤ grade 1. Key exclusion criteria included age <18, Eastern Cooperative Oncology Group performance status >2, prior anti PD-1, PD-L1, or PD-L2 therapy, history of a prior malignancy with active disease in the last 5 years with the exception of adequately treated basal cell cancer or squamous cell cancer, active central nervous system metastasis, current infection requiring antibiotic treatment, active autoimmune disease requiring treatment, hemoglobin <9.0 g/dL, absolute neutrophil count <1,500/mL, platelets <100,000/mL, or abnormal liver or kidney function. Patients with pulmonary conditions such as sarcoidosis, pneumonitis, and those with evidence of interstitial lung disease were excluded.

The study was approved by the Institutional Review Boards at the participating institutions and was conducted in accordance with the International Conference on Harmonization Good Clinical Practice guidelines and all applicable local regulatory requirements and laws. All patients provided written informed consent. The trial was registered at clinicaltrials.gov (NCT02549209). Coordination and monitoring of trial activities was provided by the BTCRC and the Lurie Cancer Center Data Safety and Monitoring Board reviewed clinical trial activities every 6 months. CONSORT guidelines were followed for trial reporting.

### Study Procedures and Endpoints

Tumor assessments were completed at baseline, at 9 weeks (before cycle 4), at the completion of treatment (∼18 weeks), and then every 12 weeks for the first year. Surveillance was performed every 3 months until progression or up to 18 months. The primary endpoint of this study was ORR as defined by the proportion of evaluable subjects with a partial (PR) or complete response (CR). Patients who received at least one dose of the study drug and underwent posttreatment radiographic assessment were considered evaluable. Secondary objectives included PFS, duration of response, and toxicity. Tumor responses for primary and secondary endpoints were assessed by the investigator per immune-related RECIST (irRECIST; refs. 28, 29). Patients were censored for PFS at the time of the last irRECIST evaluation or at the time of starting alternative cancer therapy. Duration of response was defined as duration between the date of the subject's first response (CR or PR) to the first of the date of first recorded progression of disease, and patients were censored at the time of last irRECIST evaluation or start of alternative cancer therapy. MMR/MSI status was assessed on the basis of local testing per institutional guidelines. Prespecified biomarker endpoints included ORR and PFS by MMR/MSI status. Adverse events (AE) were evaluated before every cycle and graded according to Common Terminology Criteria for Adverse Events version 4.0. Toxicity was managed with supportive medications, treatment interruption, dose reduction, and/or treatment discontinuation.

### Peripheral Blood Mononuclear Cell Processing

Peripheral blood was collected in heparanized Vacutainer tubes and peripheral blood mononuclear cells (PBMC) were isolated by Ficoll density centrifugation. Cells were resuspended in freezing media, shipped to a central location, and cryopreserved in liquid nitrogen. Characterization of circulating immune cell populations were evaluated in blood samples by Cytek Aurora.

### Spectral Cytometry CyTEK Staining and Data Analysis

For Cytek staining, cells were incubated in Fc blocker for 10 minutes at room temperature, followed by the viability staining for 15 minutes at room temperature. Cells were washed and incubated with surface staining antibody cocktail for 30 minutes at 4°C. Cells were washed and resuspended in PBS for acquisition on Aurora. The data analysis for CyTEK was performed as described previously (30). Briefly, the FCS files generated were manually gated to live CD45^+^ cells, downsampled and sequentially gated for the merged datasets using FlowJo. Clustering analyses were performed using the viSNE (31), and FlowSOM (32) algorithms within FlowJo and OMIQ web applications per the developers’ instructions. All events were sampled with a minimum estimated cluster size of 1% (∼1,000 events). The Significance Analysis of Microarrays (SAM) association model (33) was used for clustering analysis. For the differential analyses, we used the edgeR method (34). Select significant FlowSOM clusters were plotted onto the viSNE map for visualization.

### PD-L1 Staining

Archival paraffin embedded tumor tissue was stained at Qual Teck by using the Merck 22C3 antibody. PDL1 expression was scored by a board-certified pathologist. Evaluable samples had to have at least 50 viable cancer cells or at least five viable PDL1 staining cells. An H score was calculated as the product bet-ween PD-L1 membrane staining intensity (0 to 3+) and percentage of cancer cells staining (range, 0–300). PD-L1 expression was considered “positive” if the H score was >0 and “negative” if the H score was 0.

### Statistical Methods

This was a multi-institutional phase II study with a 6-subject safety lead in portion. On the basis of the results of GOG177 and GOG209 as well as a retrospective analysis of patients receiving platinum-based chemotherapy after prior platinum therapy, a response rate of 50% was estimated for platinum-based doublet therapy in this population of both recurrent and newly diagnosed patients (6, 7, 35). A meaningful clinical response would be an improvement in the ORR by 15%, and a sample size *n* = 46 subjects provided 80.3% power to detect such a difference using a one-sided Wald test with 10% type I error rate. ORR was estimated and reported with the exact Clopper–Pearson confidence and compared with the historic control rate of 50% using a one sample exact binomial test. All patients who received treatment were assessed for safety, and AEs are reported using descriptive statistics. PFS was estimated by using the method of Kaplan–Meier and groups were compared using the log-rank test. Statistical analyses were performed using R statistical software (36). Fisher exact test correlated PD-L1 staining with response to treatment.

### Data Availability Statement

The data generated in this study are available upon request from the corresponding author.

## Results

### Patients

A total of 46 eligible patients with newly diagnosed or recurrent metastatic endometrial cancer were enrolled between September 18, 2017 and December 12, 2019 at six collaborating Big Ten Cancer Research Consortium centers (CONSORT Diagram; Supplementary Fig. S1). All patients were evaluated for safety and PFS endpoints. The first 6 enrolled patients completed the safety lead-in portion of the trial at the planned dosing without experiencing a DLT, thus no dose modifications to the starting doses of the regimen were required.

Demographic and clinical characteristics of enrolled patients are reported in Table 1. Median age was 66 (range, 43–86) and the majority of enrolled patients were White (84.8%). Most patients had recurrent disease (73.9%), and either endometrioid (58.7%) or serous (23.9%) histology. Tumor grade was relatively evenly distributed between grade 1 (30.4%), grade 2 (21.7%), and grade 3 (47.8%). MMR/MSI status was available for 40 of the enrolled patients; 9 patients (22.5%) were dMMR/MSI-H and 31 patients were pMMR/MSS (77.5%). Nineteen patients had received prior carboplatin/paclitaxel, 23 women had received prior pelvic external beam radiotherapy (EBRT), 14 patients had received prior brachytherapy, 1 patient had prior doxorubicin and 1 had prior hormonal therapy.

**TABLE 1:** Patients’ characteristics (*n* = 46)

Age	
Mean (SD)	66.5 (8.9)
Median (range)	66.0 (43.0–86.0)
Race	
White	39 (84.8%)
Black or African American	3 (6.5%)
Asian	1 (2.2%)
Unknown	3 (6.5%)
Grade	
1	14 (30.4%)
2	10 (21.7%)
3	22 (47.8%)
Diagnosis type	
Primary	12 (26.1%)
Recurrent	34 (73.9%)
Histology	
Clear cell	3 (6.5%)
Endometrioid	27 (58.7%)
Mucinous	0 (0.0%)
Other	5 (10.9%)
Serous	11 (23.9%)
Previous treatment	
Carboplatin/paclitaxel	19 (41.3%)
External beam radiotherapy	23 (50.0%)
Brachytherapy	14 (30.4%)

### Efficacy

**FIGURE 1:**
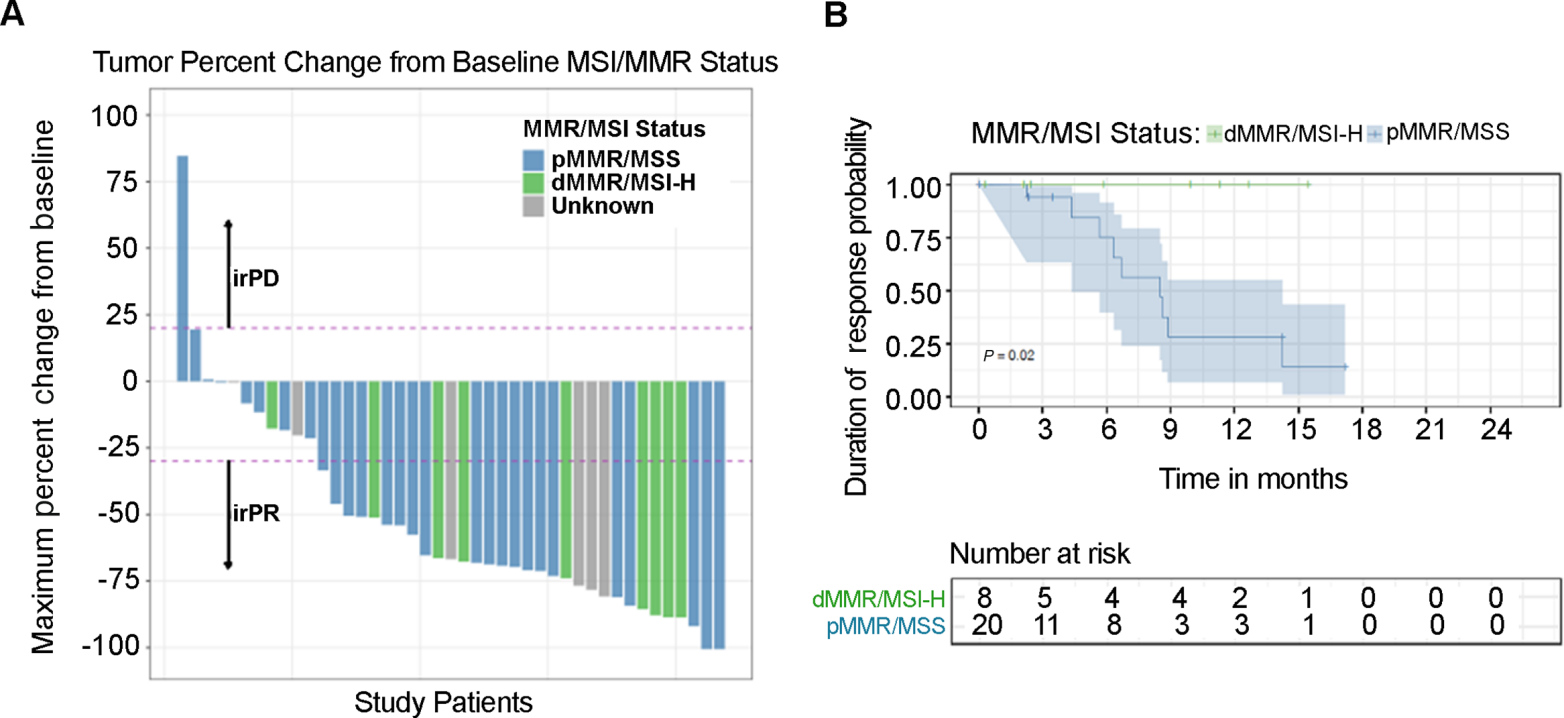
Objective responses to treatment: Tumor percent change from baseline by MMR/MSI status (*n* = 40; **A**). **B,** Duration of overall response by MMR/MSI status (*n* = 28).

Forty-three of the enrolled patients were evaluable for efficacy using the primary endpoint of ORR. Three patients received a single dose of therapy and were not evaluable for response due to transition to hospice care, a severe allergic reaction to paclitaxel, or no further follow up. A CR (*n* = 1) or PR (*n* = 31) was observed in 32 patients for an ORR of 74.4% [95% confidence interval (CI), 58.8–86.5]. The sum of diameters of target lesions decreased from baseline in 38 (88.3%) patients; 30 (69.8%) had a maximum decrease of ≥50% and 12 (27.9%) had a maximum decrease of ≥75% (Fig. 1A). Among patients with a response (*n* = 32), the median duration of response was 11.8 (95% CI, 6.7–not reached) months (Fig. 1B).

**FIGURE 2:**
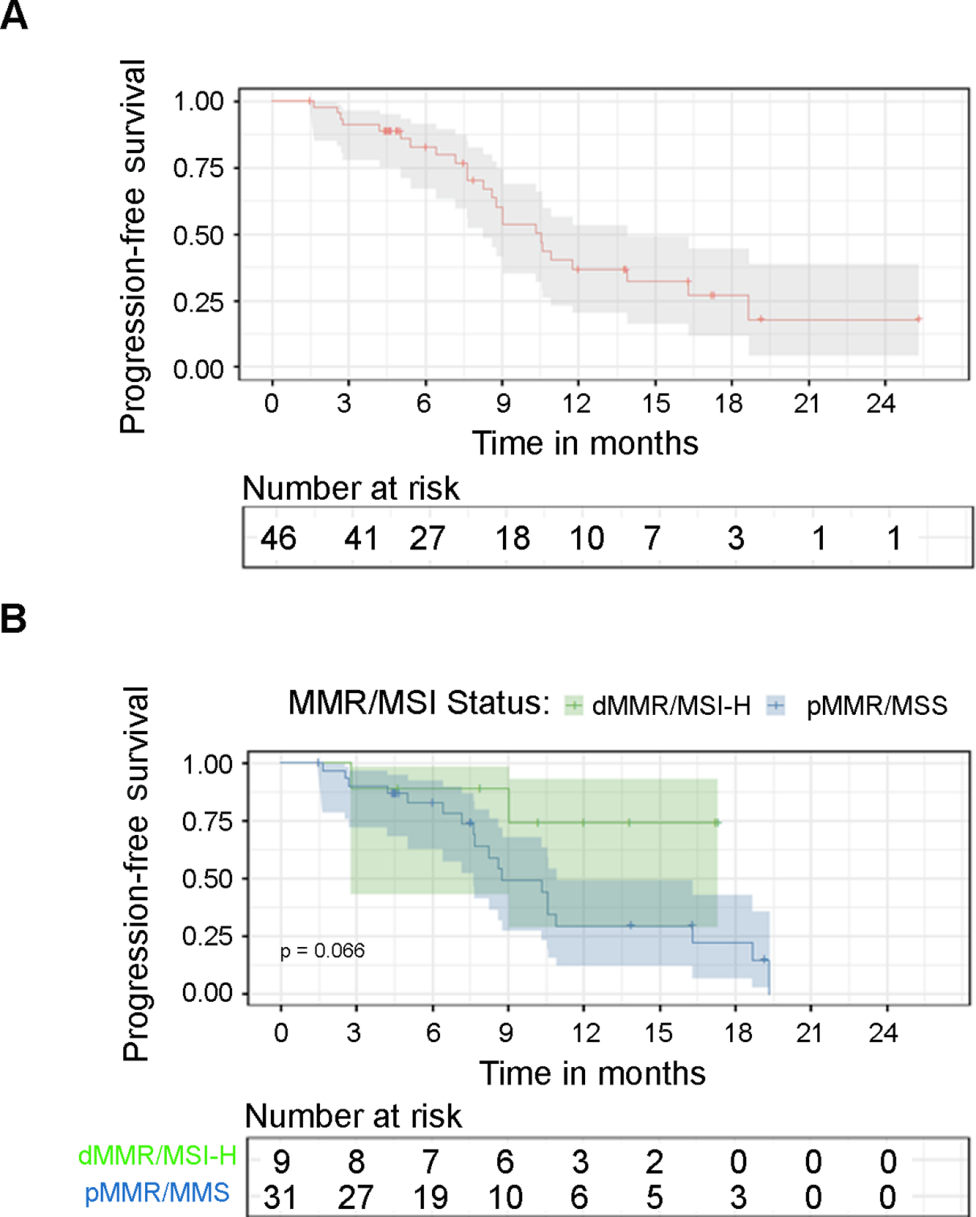
PFS: PFS was calculated by Kaplan–Meier method (*n* = 46; **A**). **B**, PFS by MMR/MSI status (*n* = 40).

**FIGURE 3:**
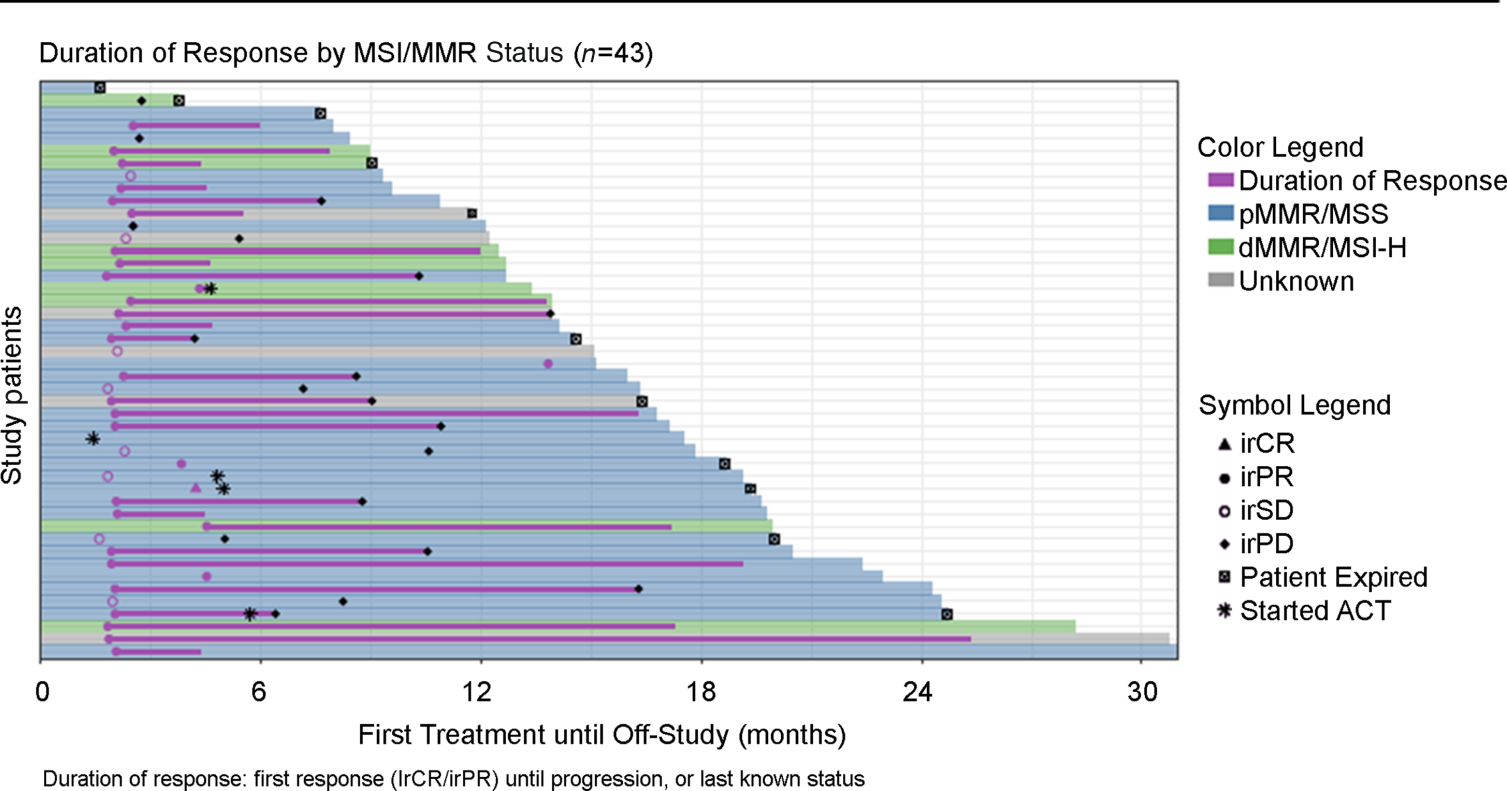
Swimmer Plot Illustrates PFS and duration of response for each patient by MMR/MSI status (*n* = 43). Green corresponds to MMR deficient, blue to MMR proficient and gray indicates unknown MMR status.

The median PFS among enrolled patients was 10.6 months (95% CI, 8.3–13.9), with 36.9% (95% CI, 20.5–53.3) of patients being progression free at 12 months (Fig. 2A). A stratified analysis was then performed by MMR/MSI status. Among *n* = 37 evaluable patients with known MMR/MSI status, ORR was 88.9% (8/9 patients; 95% CI, 51.8–99.7) among dMMR and/or MSI-H patients and 71.4% (20/28 patients; 95% CI, 51.3–86.8) among patients who were pMMR and/or MSS (*P* = 0.40). Median PFS was 8.8 months (95% CI, 1.5–16.2) for pMMR/MSS patients and was not reached for dMMR/MSI-H patients (*P* = 0.07; Fig. 2B). The median duration of response was not reached for those who were dMMR/MSI-H and was 8.5 months (95% CI, 4.37–14.3) for those who were pMMR/MSS (Fig. 3).

### Toxicity

Treatment related AEs (TRAE) occurred in 100% of patients. Serious AEs (SAE) were defined as those resulting in death, hospitalization, life-threatening or persistent incapacity or disability. SAEs were observed in 16 patients (35%), of which 9 were assessed as related to pembrolizumab [fever (*n* = 3), anaphylaxis, fatigue, hyperthyroidism, colitis, nausea, and acute kidney injury] and 9 were grade 3–4 [gastrointestinal (*n* = 3), syncope (*n* = 2), dehydration, thromboembolism, anaphylaxis, infection, fatigue, hypokalemia]. There were no grade 5 SAEs in this study. Five patients (10.9%) discontinued treatment due to an AE, as listed in Supplementary Table S1.

The most common TRAEs occurring in >5% of patients are reported in Table 2. The most common grade 1–2 TRAEs included anemia (57%), fatigue (48%), and peripheral sensory neuropathy (44%), while the most common grade 3–4 TRAEs were decreased lymphocytes, white blood cells, and anemia (each 20%). The most common grade 1–2 irAEs (Supplementary Table S2) were fatigue (39%), anemia (28%), and diarrhea (26%), and the most common grade 3–4 TRAEs were anemia (13%) and nausea, maculopapular rash and hypokalemia (4.3% each).

**TABLE 2:** Worst grade toxicity from any treatment for >5% of patients

Term	Grade 1/2 *n* (%)	Grade 3/4 *n* (%)	Overall *n* (%)
Anemia	26 (56.5%)	9 (19.6%)	35 (76.1%)
White blood cell decreased	13 (28.3%)	9 (19.6%)	22 (47.8%)
Lymphocyte count decreased	7 (15.2%)	9 (19.6%)	16 (34.8%)
Neutrophil count decreased	10 (21.7%)	6 (13%)	16 (34.8%)
Platelet count decreased	15 (32.6%)	5 (10.9%)	20 (43.5%)
Hypokalemia	6 (13%)	5 (10.9%)	11 (23.9%)
Fatigue	22 (47.8%)	2 (4.3%)	24 (52.2%)
Peripheral sensory neuropathy	20 (43.5%)	2 (4.3%)	22 (47.8%)
Nausea	17 (37%)	2 (4.3%)	19 (41.3%)
Rash maculopapular	7 (15.2%)	2 (4.3%)	9 (19.6%)
Anorexia	8 (17.4%)	1 (2.2%)	9 (19.6%)
Vomiting	7 (15.2%)	1 (2.2%)	8 (17.4%)
Hypophosphatemia	6 (13%)	1 (2.2%)	7 (15.2%)
Alkaline phosphatase increased	4 (8.7%)	1 (2.2%)	5 (10.9%)
Hypertension	3 (6.5%)	1 (2.2%)	4 (8.7%)
Dehydration	2 (4.3%)	1 (2.2%)	3 (6.5%)
Hematuria	2 (4.3%)	1 (2.2%)	3 (6.5%)
Hyponatremia	2 (4.3%)	1 (2.2%)	3 (6.5%)
Pruritus	2 (4.3%)	1 (2.2%)	3 (6.5%)
Alopecia	22 (47.8%)	0 (0%)	22 (47.8%)
Diarrhea	16 (34.8%)	0 (0%)	16 (34.8%)
Hypomagnesemia	16 (34.8%)	0 (0%)	16 (34.8%)
Constipation	11 (23.9%)	0 (0%)	11 (23.9%)
Hypothyroidism	11 (23.9%)	0 (0%)	11 (23.9%)
Arthralgia	10 (21.7%)	0 (0%)	10 (21.7%)
Dyspnea	10 (21.7%)	0 (0%)	10 (21.7%)
Myalgia	9 (19.6%)	0 (0%)	9 (19.6%)
Alanine aminotransferase increased	6 (13%)	0 (0%)	6 (13%)
Aspartate aminotransferase increased	6 (13%)	0 (0%)	6 (13%)
Cough	6 (13%)	0 (0%)	6 (13%)
Hyperthyroidism	6 (13%)	0 (0%)	6 (13%)
Abdominal pain	5 (10.9%)	0 (0%)	5 (10.9%)
Dysgeusia	5 (10.9%)	0 (0%)	5 (10.9%)
Infusion related reaction	5 (10.9%)	0 (0%)	5 (10.9%)
Pain in extremity	5 (10.9%)	0 (0%)	5 (10.9%)
Creatinine increased	4 (8.7%)	0 (0%)	4 (8.7%)
Edema limbs	4 (8.7%)	0 (0%)	4 (8.7%)
Endocrine disorders ‐ other, specify	4 (8.7%)	0 (0%)	4 (8.7%)
Fever	4 (8.7%)	0 (0%)	4 (8.7%)
Hypoalbuminemia	4 (8.7%)	0 (0%)	4 (8.7%)
Anxiety	3 (6.5%)	0 (0%)	3 (6.5%)
Dizziness	3 (6.5%)	0 (0%)	3 (6.5%)
Dry skin	3 (6.5%)	0 (0%)	3 (6.5%)
Headache	3 (6.5%)	0 (0%)	3 (6.5%)
Hypotension	3 (6.5%)	0 (0%)	3 (6.5%)
Rash acneiform	3 (6.5%)	0 (0%)	3 (6.5%)

### Immune Profiles Associated with Response

**FIGURE 4:**
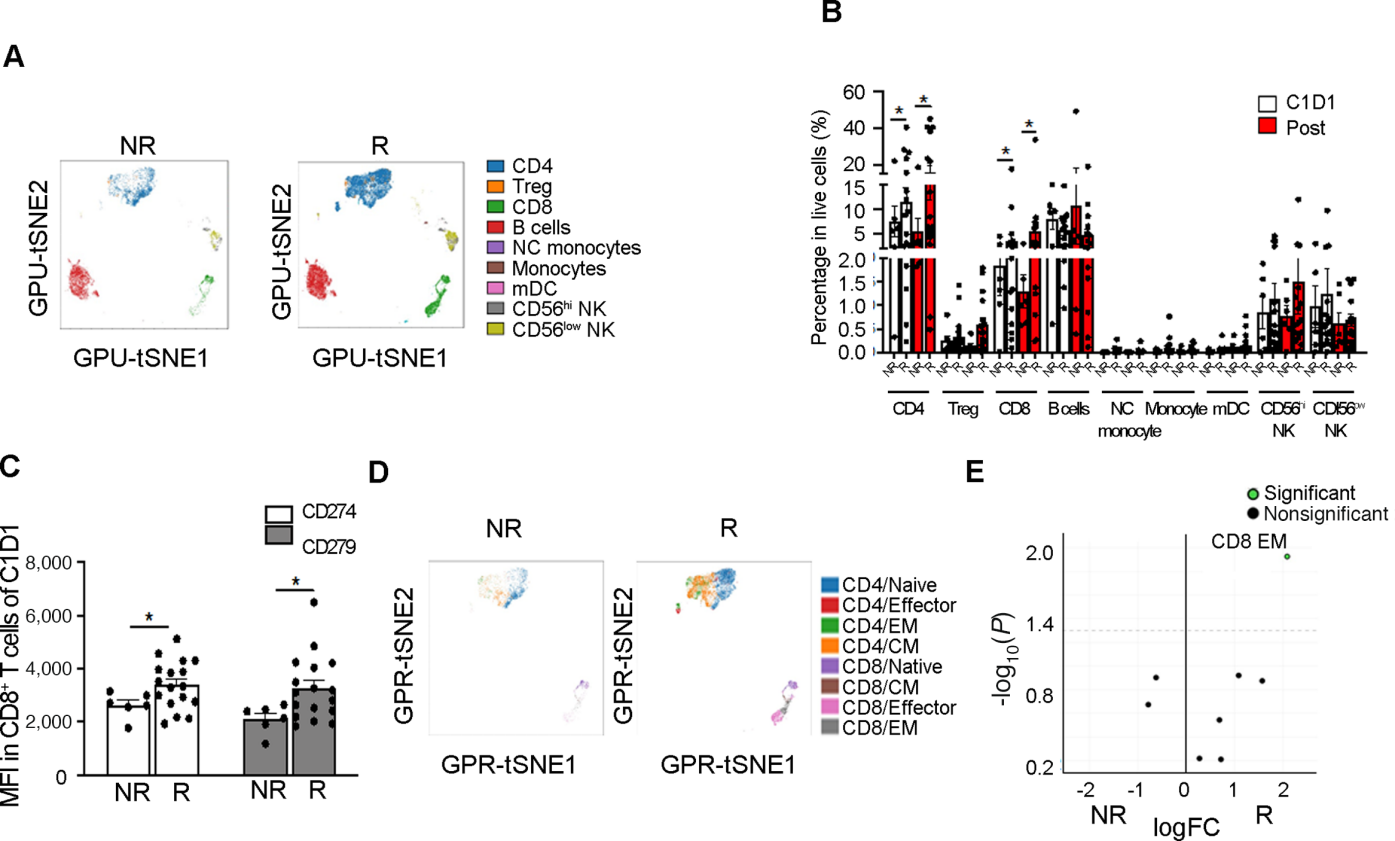
Identification of differences in PBMC subsets from responders and nonresponder patients before treatment using spectral cytometry. **A,** Exemplified GPU-tSNE visualization of overlaid cell population composition in the PBMC from nonresponders (*n* = 6) and responders (*n* = 17) before the initiation of therapy (C1D1). **B,** Summarized frequencies of total main immune cell populations among live PBMC from nonresponders or responders at C1D1 and 30 days after treatment. **C,** The expression levels of CD274 and CD279 were compared in peripheral CD8^+^ T cells between nonresponders and responders at C1D1. **D**, Exemplified GPU-tSNE visualization of overlaid memory subset composition in peripheral CD8^+^ and CD4^+^ T cells from nonresponders and responders at C1D1. **E,** EdgeR analysis identified the CD8^+^ EM cell subset with significant differences in relative abundance between nonresponders and responders at C1D1. Error bars represent mean with SEM. *, *P* < 0.05. All *P* values were calculated using two-sided *t* tests and were corrected for the multiple comparisons using the Benjamini–Hochberg adjustment.

We performed high-dimensional spectral flow cytometry (CyTEK) analysis using cryopreserved PBMC samples obtained from 23 patients enrolled in the trial before [cycle 1 day 1 (C1D1)] and 30 days after treatment. The dimensionality reduction tool tSNE by a GPU-accelerated implementation compared responders (*n* = 17) versus nonresponders (*n* = 6) at baseline, prior to starting the combination therapy. Live intact single cells gated from the C1D1 PBMC specimens could be clearly grouped into distinct subsets on the viSNE map (Fig. 4A), including CD4^+^ T cells (CD3^+^CD4^+^), regulatory T cells (CD4^+^CD25^hi^), CD8^+^ T cells (CD3^+^CD8^+^), B cells (CD19^+^CD20^+^), classic monocytes (Lin^−^CD14^+^CD16^−^), nonclassical monocytes (Lin-CD14^low^CD16^+^), monocytic dendritic cells, CD56^hi^ and CD56^dim^ natural killer (NK) cells (CD3^−^CD56^+^CD16^+^/^low^), and monocytes (CD33^+^CD14^+^). Supplementary Figure S2 includes the gating strategy. Both peripheral CD8^+^ T-cell and CD4^+^ T-cell populations at C1D1 tended to be enriched in PBMCs from responders as shown by the viSNE map (Fig. 4A). Consistently, the frequencies of both T-cell populations were significantly higher in responders than in nonresponders at baseline (C1D1), as estimated by using conventional supervised gating on FlowJo (Fig. 4B). Although the frequencies of all the major immune subsets did not show significant differences between C1D1 and posttreatment, the elevated frequencies of both CD4 and CD8 T-cell populations were also observed in posttreatment samples in responders compared with nonresponders (Fig. 4B). By examining in detail the functional phenotypes of differentially abundant immune cell populations, we observed that responders exhibited greater expression levels of PD-1 (CD279), and PD-L1 (CD274) in CD8^+^ T-cell compartments than nonresponders at C1D1 (Fig. 4C). Furthermore, we examined the CD4 and CD8 T-cell populations by using CD45RA, CD27, CD28, and CCR7 additional cell surface markers to define naïve (CD45RO^−^CD45RA^+^CD27^+^CD28^+^CCR7^+^), central memory (CD45RO^+^CD45RA^−^CD27^+^CD28^+^CCR7^+^), effector memory (EM, CD45RO^+^CD45RA^−^CD27^+/−^ CD28^+/−^CCR7^−^), and terminally differentiated effector memory (CD45RO^−^CD45RA^+^CD27^−^ CD28^−^CCR7^−^) cells. The differential abundance of these differentiated T-cell subsets determined on the basis of conventional supervised gating above was visualized by tSNE (Fig. 4D) and further analyzed using the edgeR package (Fig. 4E). Despite the relatively large variability observed within each group, CD8^+^ EM cells were statistically more abundant in responders than that in nonresponders at baseline, prior to start of therapy (Fig. 4E).

**FIGURE 5:**
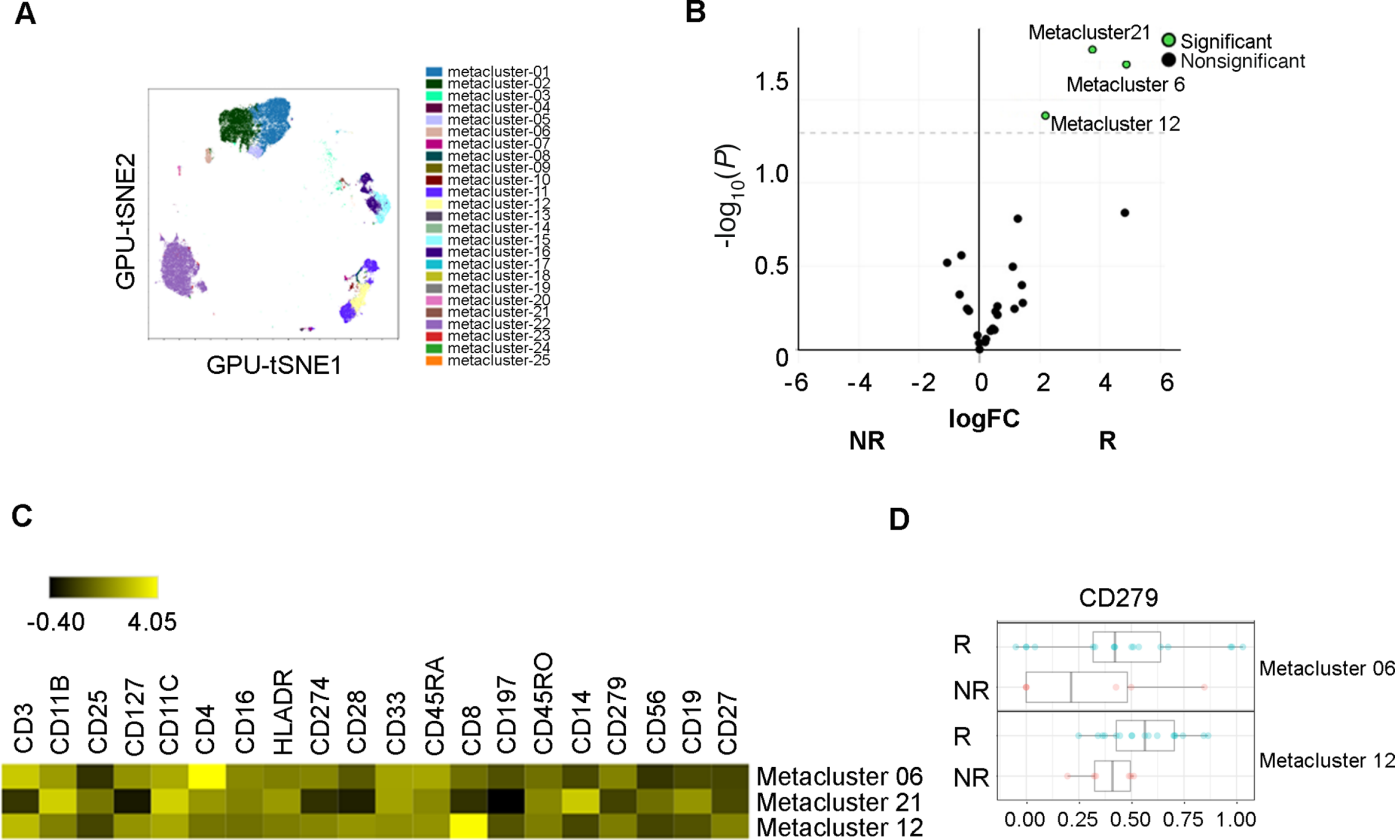
Identification of metaclusters in PBMC with significant differences between responders and nonresponders before treatment. **A,** Exemplified GPU-tSNE visualization of overlaid unsupervised metaclusters in PBMC using the FlowSOM algorithm from nonresponders (*n* = 6) and responders (*n* = 17) at C1D1 prior to therapy. **B,** EdgeR analysis identified metaclusters with significant differences in relative abundance between nonresponders and responders. **C,** The heat map represents the median expression levels of indicated markers within the metaclusters having significant differences in relative abundance between nonresponders and responders at C1D1. **D,** Relative expression levels of CD279 in the metaclusters of 6 and 12 were significantly different between nonresponders and responders at C1D1 based on the SAM analysis (*P*_adjusted_ < 0.05).

To minimize investigator-associated biases and variability related to supervised manual analysis of cytometry data, we next used a minimally supervised, standardized analytical workflow based on the FlowSOM algorithm and the dimensionality reduction tool GPU-tSNE (Fig. 5A), in addition to the conventional manual gating and supervised analysis described above. Live, intact single cells were clustered using cell surface markers into a FlowSOM tree using unsupervised clustering to yield 25 metaclusters (Supplementary Fig. S3). The edgeR analysis (Fig. 5B) identified an increase in clusters 6, 12, and 21 in responders compared with nonresponders (Fig. 5C) at C1D1. The cluster 21 represented a subset of CD3^−^CD14^+^CD16^low^ intermediate and/or classical monocyte-like population that express HLA-DR, CD33, CD11b, and CD11c. The clusters 6 and 12 were among CD3^+^CD4^+^ T-cell and CD3^+^CD8^+^ T-cell populations, respectively, both positive for CD45RO and CD127 with dim or null expression of CD197 (CCR7; Fig. 5D), indicating a T-cell EM subset but reexpressing CD45RA. Interestingly, the cluster 12 was further positive for CD27 and CD28, suggesting a relatively less differentiated state (compared with the cluster 6 with dim or null expression of CD27 and CD28 (Fig. 5C). Moreover, both EM subsets (the clusters 6 and 12) displayed higher levels of PD-1 (CD279) at C1D1 in responders than those in nonresponders. Collectively, the results of combined supervised and unsupervised multidimensional analyses indicate distinct circulating populations of EM T-cell subsets enriched in responders at baseline.

### PD-L1 Staining

Archival tissue from 31 enrolled patients was available and stained for PD-L1; 18 specimens (58%) were positive (H-score range, 1–195) and 13 (42%) were negative (H score 0). Surprisingly, there were more PD-L1–expressing tumors among nonresponders than among responders (*P* = 0.025; Supplementary Table S3).

## Discussion

Advanced endometrial cancer is a deadly disease for which treatment has evolved slowly compared with other solid tumors. Here we provide evidence that addition of anti-PD-1 treatment to the standard combination carboplatin and paclitaxel is well tolerated and active, inducing a response rate that exceeded historical results (6, 7). We also identify biomarkers in the peripheral circulation associated with clinical response. Our data support further testing of this combination in a randomized manner compared with standard chemotherapy.

Current treatment options for metastatic or recurrent endometrial cancer consists of carboplatin and paclitaxel (ORR 52%), lenvatinib and pembrolizumab (ORR 38%), pembrolizumab alone for dMMR (ORR 57%) as well as carboplatin/paclitaxel with trastuzumab for Her-2/neu positive serous endometrial cancer (6, 16, 18, 37). The study presented here met its primary endpoint with a response rate of 74.4%, exceeding outcomes expected with the currently available treatment strategies, although the median PFS recorded here was similar to that noted in previous studies. This was observed despite the high-risk nature of the study population, including a majority of patients with recurrent disease, previously treated with chemotherapy or EBRT, and nearly a quarter of patients with highly aggressive serous endometrial cancer. By comparison, GOG-209 and GOG-177 protocols that tested chemotherapy alone, restricted enrollment to patients who had not received prior chemotherapy and reported lower ORR and similar PFS (6, 7). It is possible that lack of a maintenance treatment phase contributed to the lack of response durability and similar PFS compared with previous studies. The use of maintenance anti-PD1 treatment to extend response duration should be investigated in future studies. The safety profile and tolerability of the three-agent combination was overall acceptable with 10.9% of patients discontinuing therapy due to AEs. Known toxicities reported for carboplatin, paclitaxel, and pembrolizumab were observed at expected rates for this population. There were no new safety signals or increase in frequencies of observed toxicities attributable to the combination therapy.

Most patients with endometrial cancer have pMMR/MSS tumors (38). There are significant differences in response rates to single-agent anti-PD1/PDL1 therapies between patients with dMMR/MSI-H and those with pMMR/MSS endometrial cancers. Response rates for pMMR tumors range from 3% to 19%, whereas those for dMMR tumors range from 27% to 57% (17, 39–41). In this study, we observed a difference between the ORR in pMMR/MSS tumors (71.4%) versus dMMR/MSI-H tumors (89.9%); however, this did not reach statistical significance given the low numbers of patients in the two subgroups. The ORR of 71.4% in pMMR/MSS tumors exceeded the response rate to carboplatin and paclitaxel alone (18); however, median DOR was only 8.5 months. In contrast, responses were sustained and median PFS was not reached in the dMMR/MSI-H group.

Presence of PD-L1 expression did not corelate positively with clinical response in this patient group; in fact more patients with negative PD-L1 tumors responded to treatment. Interestingly, previous reports have also not detected a correlation between response to immune checkpoint blockade and PDL1 expression in endometrial cancer (40). This is not surprising as it is likely that a compartmentalized analysis of PD-L1 expression within tumors is required to confer prognostic significance (42). Our results suggest that pembrolizumab in combination with chemotherapy is an effective strategy even for MSS tumors and PD-L1–negative tumors.

Little is known about potential biomarkers to monitor or predict patients’ responses to immunotherapies in prospective endometrial cancer cohort studies (43, 44). Current studies on exploring prognostic and immune-related gene signatures in endometrial cancer mainly concentrate on analyses in respective database in public domain (e.g., The Cancer Genome Atlas) by applying bioinformatics. Using comprehensive immune monitoring, we observed the elevated frequencies of peripheral conventional T-cell subsets at baseline and posttreatment in patients with endometrial cancer with a clinical response to chemotherapy in combination with pembrolizumab compared with those in nonresponders. Furthermore, in responding patients, we found higher frequencies of EM subsets reexpressing CD45RA (TEMRAs) from both CD8 and CD4 T-cell populations prior to start of treatment. On the basis of these results, series cytometric examination of the number and memory state of peripheral blood conventional T cells might be developed into a biomarker to predict the response to chemotherapy in combination with pembrolizumab in endometrial cancer.

Our findings are supported by several studies investigating peripheral memory T-cell subsets, particularly in relation to immune checkpoint inhibitor (ICI) treatment in tumor types other than endometrial cancer. In non–small cell lung cancer (NSCLC), malignant pleural mesothelioma (45) and melanoma (46), EM-like CD4 T cells (47) and/or CD8 TEMRA at baseline were associated with response to ICI. Notably, we found higher levels of PD-1 and/or PD-L1 especially on peripheral CD8 TEMRA lacking costimulatory receptors CD27 and CD28 in responders than those in nonresponders, indicating these cells are potentially susceptible to reinvigoration by combined anti-anti-1/PD-L1 ICI treatment as these cells likely retain their proliferative capacity and the ability to infiltrate to the tumor upon treatment (48, 49). Along these lines, Kunert and colleagues reported that the frequency of peripheral CD8 T cells devoid of multiple costimulatory receptors was highest in PR patients with NSCLC at baseline and throughout anti-PD-1 therapy and correlated with the total number of CD8 T cells as well as frequencies of CD8 TEMRA phenotype (50). Several other studies have also shown prolonged survival outcomes and positive prognostic feature in cases with high baseline PD-1^+^CD8^+^ cells (48, 51), which is in contrast with the presence of intratumoral PD-1^+^CD8^+^ counterparts that often display exhausted phenotype linked to unfavorable outcomes across a range of human cancers (52, 53). Our data suggest that peripheral blood analysis may provide valuable insights into predicting responses to PD-1–targeted therapies in endometrial cancer and warrant further studies on identification of peripheral blood biomarkers using larger and independent patient cohorts with attention to immune alterations within tumoral compartment.

The study was limited by its nature as a single-arm phase II study with no comparator group. In addition, the PFS achieved with the combined regimen was similar to the PFS recorded in trials testing chemotherapy alone, albeit those studies targeted less heavily pretreated populations. However, the results of the current trial provide confidence that randomized studies testing similar chemoimmunotherapy regimens will achieve significance and perhaps lead to a new standard for first-line treatment of metastatic endometrial cancer. Several placebo-controlled randomized phase III studies NRG-018 (NCT03914612), RUBY/ENGOT-EN6 (NCT03981796), AtTEnd (NCT03603184), and MITO END-3 (NCT03503786) testing chemotherapy together with immune checkpoint blockade are ongoing, with results expected in the next couple of years. As we await their results, the data from this initial trial testing the chemoimmunotherapy combination provide confidence that this regimen will shape the future treatment of both newly diagnosed advanced stage and recurrent metastatic endometrial cancer.

## Supplementary Material

Supplementary Tables 1-3, Figures 1-3 Supplementary Table 1. Treatment discontinuation due to AE. Supplementary Table 2. Worst grade toxicity related to pembrolizumab in >=5% of patients. Supplementary Table 3. PDL1 staining by objective responder status. Supplementary Figure S1. CONSORT diagram illustrates distribution of patients after enrollment. Supplementary Figure S2. Gating strategies for the main immune populations in PBMC. Supplementary Figure S3. The FlowSOM tree for the PBMC collected at C1D1 showing unsupervised metaclustering.
