# Supplementary material for: Clinical and Biological Activity of Chemoimmunotherapy in Advanced Endometrial Adenocarcinoma: A Phase II Trial of the Big Ten Cancer Research Consortium
Source: Cancer Res Commun. 2022 Oct 28;2(10):1293–303. doi: 10.1158/2767-9764.CRC-22-0147 (PMC9648489; doi:10.1158/2767-9764.CRC-22-0147)

**Supplementary Material:**

Supplementary Table 1. Treatment discontinuation due to AE

| ID | Treatment discontinuation due to AE | Category | Term |
| --- | --- | --- | --- |
| B013‐1001 | Yes | Metabolism and nutrition disorders | Dehydration |
| B013‐1007 | Yes | General disorders and administration site conditions | Infusion related reaction |
| B013‐1024 | Yes | Respiratory, thoracic and mediastinal disorders | Dyspnea |
| B013‐1027 | Yes | Immune system disorders | Anaphylaxis |
| B013‐1028 | Yes | Nervous system disorders | Peripheral sensory neuropathy |

**Supplementary Table 2: Worst grade toxicity related to pembrolizumab in >=5% of patients**

| Term | Grade 1/2 n(%) | Grade 3/4 n(%) | Overall n(%) |
| --- | --- | --- | --- |
|  |  |  |  |
| Anemia | 13 (28.3%) | 6 (13%) | 19 (41.3%) |
| Nausea | 8 (17.4%) | 2 (4.3%) | 10 (21.7%) |
| Rash maculo‐papular | 7 (15.2%) | 2 (4.3%) | 9 (19.6%) |
| Hypokalemia | 2 (4.3%) | 2 (4.3%) | 4 (8.7%) |
| Fatigue | 18 (39.1%) | 1 (2.2%) | 19 (41.3%) |
| Anorexia | 7 (15.2%) | 1 (2.2%) | 8 (17.4%) |
| Alkaline phosphatase increased | 2 (4.3%) | 1 (2.2%) | 3 (6.5%) |
| Hematuria | 2 (4.3%) | 1 (2.2%) | 3 (6.5%) |
| Hypophosphatemia | 2 (4.3%) | 1 (2.2%) | 3 (6.5%) |
| White blood cell decreased | 2 (4.3%) | 1 (2.2%) | 3 (6.5%) |
| Diarrhea | 12 (26.1%) | 0 (0%) | 12 (26.1%) |
| Hypothyroidism | 11 (23.9%) | 0 (0%) | 11 (23.9%) |
| Dyspnea | 10 (21.7%) | 0 (0%) | 10 (21.7%) |
| Constipation | 7 (15.2%) | 0 (0%) | 7 (15.2%) |
| Cough | 6 (13%) | 0 (0%) | 6 (13%) |
| Hyperthyroidism | 6 (13%) | 0 (0%) | 6 (13%) |
| Hypomagnesemia | 6 (13%) | 0 (0%) | 6 (13%) |
| Abdominal pain | 5 (10.9%) | 0 (0%) | 5 (10.9%) |
| Alanine aminotransferase increased | 5 (10.9%) | 0 (0%) | 5 (10.9%) |
| Arthralgia | 5 (10.9%) | 0 (0%) | 5 (10.9%) |
| Aspartate aminotransferase increased | 5 (10.9%) | 0 (0%) | 5 (10.9%) |
| Platelet count decreased | 5 (10.9%) | 0 (0%) | 5 (10.9%) |
| Dysgeusia | 4 (8.7%) | 0 (0%) | 4 (8.7%) |
| Endocrine disorders ‐ other, specify | 4 (8.7%) | 0 (0%) | 4 (8.7%) |
| Peripheral sensory neuropathy | 4 (8.7%) | 0 (0%) | 4 (8.7%) |
| Creatinine increased | 3 (6.5%) | 0 (0%) | 3 (6.5%) |
| Dizziness | 3 (6.5%) | 0 (0%) | 3 (6.5%) |
| Fever | 3 (6.5%) | 0 (0%) | 3 (6.5%) |
| Headache | 3 (6.5%) | 0 (0%) | 3 (6.5%) |
| Hypoalbuminemia | 3 (6.5%) | 0 (0%) | 3 (6.5%) |
| Myalgia | 3 (6.5%) | 0 (0%) | 3 (6.5%) |
| Pain in extremity | 3 (6.5%) | 0 (0%) | 3 (6.5%) |
| Rash acneiform | 3 (6.5%) | 0 (0%) | 3 (6.5%) |
| Vomiting | 3 (6.5%) | 0 (0%) | 3 (6.5%) |

**Supplementary Table 3: PDL1 staining by objective responder status:**


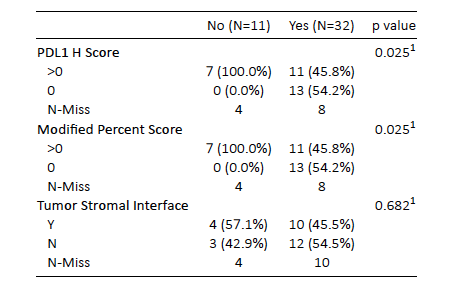


**Supplementary Figures**

Figure S1: CONSORT diagram illustrates distribution of patients after enrollment


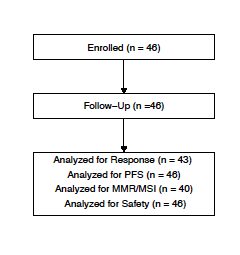


**Supplementary Figure S2:** **Gating strategies for the main immune populations in PBMC.** Single cell suspensions of PBMC were stained and analyzed by CyTEK for measuring CD4^+^ T cells (CD3^+^CD4^+^), CD8^+^ T cells (CD3^+^CD8^+^), B cells (CD19^+^CD3^-^), classic monocytes (Lin^-^CD14^+^CD16^-^), non-classical (NC) monocytes (Lin^-^CD14^low^CD16^+^), monocytic dendritic cells (mDC), CD56^hi^ and CD56^dim^ NK cells.

**Supplementary Figure S3:** **The FlowSOM tree for the PBMC collected at C1D1 showing unsupervised metaclustering.**The background coloring indicates the relative abundance of each metacluster.  FlowSOM tree with unsupervised clustering


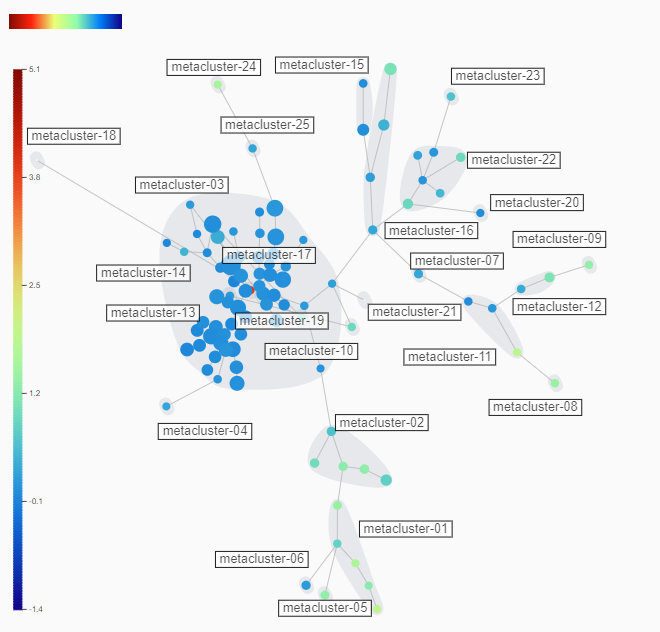

Supplement: Supplementary Tables 1-3, Figures 1-3 — Supplementary Table 1. Treatment discontinuation due to AE. Supplementary Table 2. Worst grade toxicity related to pembrolizumab in >=5% of patients. Supplementary Table 3. PDL1 staining by objective responder status. Supplementary Figure S1. CONSORT diagram illustrates distribution of patients after enrollment. Supplementary Figure S2. Gating strategies for the main immune populations in PBMC. Supplementary Figure S3. The FlowSOM tree for the PBMC collected at C1D1 showing unsupervised metaclustering. [file crc-22-0147-s01.docx]
